# Fake smoke, real fire: a retrospective single-center analysis of the underestimated risk of e-cigarette explosions and the typical burn patterns

**DOI:** 10.1007/s00508-025-02520-y

**Published:** 2025-04-29

**Authors:** Christian Freystätter, Clement Staud, Anna Fast, Philipp Tratnig-Frankl, Gerald Ihra, Christine Radtke

**Affiliations:** 1 Department of Plastic, Reconstructive and Aesthetic Surgery, Medical University of Vienna, Vienna, Austria; 2 Department of Anesthesiology and General Intensive Care, Medical University of Vienna, Vienna, Austria

**Keywords:** Smoking, Burn unit, Plastic surgery, Trauma, Skin graft

## Abstract

Parallel to the increasing dissemination of electronic cigarettes (E-cigarette) in the population, increasing numbers of burn injuries caused by explosions of the devices or the rechargeable lithium-ion batteries have been observed worldwide. Several cases of E‑cigarette-related explosions have already resulted in fatalities. We report a series of 7 patients who were treated at our department between 2016 and 2022. Of the seven patients two required treatment in the intensive care unit (ICU), two further patients required normal ward and three patients could be treated as outpatients. The median age of the patients was 43 years and the female to male ratio 2:5. Most of the patients had superficial to full thickness burn injuries on their thighs and (dominant) hands; however, injuries to the genitals were also observed. The patients’ burns had a median total body surface area of 4.5%. Of these patients 4 had to stay in hospital for a median of 12.5 days, 2 of them required surgical treatment and intensive care. No patient died during hospitalization. Explosions of E‑cigarettes often happen in public while the devices or rechargeable lithium-ion batteries are being carried in trouser pockets, as shown in our study. This often ignites the victim’s clothing. In a crowd or on public transport, this fire could spread quickly and endanger surrounding persons. It is therefore in the public interest to raise awareness of this potential danger from E‑cigarettes.

## Introduction

In the years 2015–2017 over 2000 burn injuries were attributed to explosions caused by explosions of electronic cigarettes (E-cigarettes) in the USA and approximately one quarter of the patients had to be hospitalized because of the severity of the injury [1]. A rising number of burns caused by E‑cigarettes up to 2022 has been described [2]. In addition to burn injuries, E‑cigarette explosions can also cause severe facial injuries and loss of body parts [3]. Media reports about the first death caused by an exploding E‑cigarette, that of a young American man in Florida in May 2018, were the impetus for the current retrospective study [4, 5].

In 1965, a patent was registered for the first time for a device that vaporized nicotine using electric current by Herbert A. Gilbert (Pennsylvania); the device was proposed as an alternative to conventional cigarettes that would be less detrimental to health [6]; however, a few decades had to pass before this invention reached the broader public. The first commercial version, known today as the E‑cigarette, was finally developed and released in 2003 by the Chinese pharmacologist Hon Lik [7]. As E‑cigarettes soon proved capable of racking up large sales numbers in China in a short time, the novel devices were introduced to the European and American markets in 2007, with a delay of approximately 3 years and were soon a great sales success [8]. Since then, E‑cigarettes have rapidly gained popularity among smokers and nonsmokers worldwide. According to recent surveys, approximately 2.75 million US citizens in 2016 and 7.5 million Europeans in 2014 have regularly consumed the aerosols generated by E‑cigarettes [9, 10]. Worldwide, approximately 450 different brands of products with different model configurations and accessories are sold and distributed via web stores or traditional businesses [11]. Independent of the model, E‑cigarettes follow a schematically similar structure and have an energy source, a voltage regulator with or without a microprocessor, a heating or vaporizing unit (atomizer), a (liquid) tank, a mouthpiece and a housing [12]. With increasing dissemination and use of these devices, however, the number of reported injuries to consumers by E‑cigarettes overheating, catching fire or exploding has also increased [13]. Such thermal incidents can occur during operation, when idle or while the E‑cigarette is being charged [11]. In the years 2015–2017, there were an estimated more than 2000 burn injuries caused by explosions of E‑cigarettes in the USA [1]. In most cases, the victim’s thighs and hands were affected, and approximately one quarter of the patients had to be hospitalized [1]. In the majority of cases reported, these injuries were caused by thermal runaway of rechargeable lithium-ion batteries (Li-ion batteries) and a resulting explosion [9, 11, 14, 15]. During a thermal runaway, the entire stored energy of the Li-ion battery is released in just a few seconds through a rapid exothermic chain reaction. In this reaction, the battery overheats, causing hot gases to develop, which are then expelled explosively as hot steam and flames. In this process, other corrosive substances, such as hydrofluoric acid, can be released [16]. Along with burn injuries and explosive trauma, thermal malfunctions of E‑cigarettes can therefore also cause acid burns on the body surface and in the airways [17, 18]. This kind of thermal runaway of a Li-ion battery can be irreversibly initiated by damage, improper use, incorrect charging, short circuit or technical defects.

According to recent scientific studies, patients typically suffer burns over less than 5% of their total body surface area (TBSA) after thermal malfunctions of E‑cigarettes. Moreover, the victims are mostly male (approximately 90%) and between 20 and 40 years of age [11, 18–21].

The aim of the present study was to determine the number of patients treated by our department for E‑cigarette-associated injuries per year, mechanism of injury, to identify accident-specific patterns of burn injuries and to evaluate the courses of treatment as well as the patient outcomes. Based on this, possible measures to reduce such incidents are reflected and discussed with the current literature.

## Material and methods

A descriptive retrospective single-center analysis of all electronic patient files with burn injuries who underwent treatment by the Department for Plastic and Reconstructive Surgery with the Center for Severe Burn Patients at the Vienna General Hospital between January 2016 and December 2022 was carried out. Patients younger than 16 years were excluded. Patients with burn injuries owing to incidents with E‑cigarettes were included in this study. A positive vote was issued by the Ethics Committee of the Medical University of Vienna (No.: 2088/2020). The patient files and photographic documentation of these patients were retrieved and studied. The patient consent was required for the photographic documentation. Patient data, the course of accidents, the type, extent and location of the wounds, the course of treatment and the outcomes were documented, collected and stored pseudonymized using the program Excel 2016 (Microsoft; Redmond, WA, USA). The extent of the total burned body surface area was calculated by the software system BurnCase 3D (RISC Software, Hagenberg, Austria). Statistically, the data were analyzed using SPSS Statistics 24.0 (IBM; Armonk, NY, USA). The demographic data, accident details, burn classification, clinical course and treatment were analyzed and described statistically. Under the assumption of a nonnormal distribution of the underlying data, metric and ordinal scaled variables are described as medians and ranges, and nominal scaled data are described as frequencies and percentages.

## Results

In total, 7 patients (*n* = 7) with burn injuries owing to explosions of E‑cigarettes or the Li-ion-rechargeable batteries who underwent treatment by our department between January 2016 and December 2022 were identified. On average, one patient per year with burn injuries related to E‑cigarette explosions was treated by our clinic. A summary of the cases is given in Table 1. An average of 75 burn patients per year received initial treatment in the intensive care unit (ICU). Compared to the overall presentation of patients, those with E‑cigarette burns is a small group.

**Table 1:** Shows the summary of the cases describing for each patient the gender, TBSA, ABSI, burn degree, anatomical location, setting of the incident, secondary diagnosis, received treatment, number of operations, length of hospital and ICU stay and the wound outcome

Case	Age [yrs]	Sex	TBSA [%]	ABSI	Full thickness burns	Body sites	Accident site	Accident course	Secondary diagnosis	Treatment	Number of Operations	Length of hospital stay [d]	Length of ICU stay [d]	Outcome
1	23	Female	9	5	Yes	Upper thigh (left, semicircular, IIb–III°), buttock (IIa°), hip (left, I–IIa°), hands (both, IIa°)	Public, bar	The patient had been sitting on the E‑cigarette, which was in the hip pocket. The E‑cigarette exploded, and the pants caught fire	None	Admitted to the ICU; Week 1–2: Four debridements (dressing with PuF-Ag) in SA (patient refused the recommended necrosectomy). Systemic antibiotics, intermediately. Week 3: Operation (thigh) tangential necrosectomy under ITN, defect coverage with ASTSG (mesh ratio 1.5:1), dressed with a VAC-system for 5 days. Conservative treatment of the other injured areas with PuF-Ag dressings. Custom-made burn pressure garments	5	31	15	The skin grafts were completely healed in place. All other burn wounds completely healed using conservative treatment. Discoloration and hyperesthesia remained
2	43	Male	8	5	Yes	Upper thighs (right, IIb–III° > left, IIa°), genital (IIa–IIb°), mons pubis (IIa–IIb°), hands (both, IIa°), abdomen (IIa°) (Fig. 1a, b)	Public, walking down the sidewalk	The Li-ion battery of the E‑cigarette (which was carried in the right pants pocket with a metallic key ring) exploded without any warning	None	Admitted to the ICU. Day 2: Debridement, re-evaluation of the wounds (dressing with PuF-Ag) under SA. Day 5: Operation (right thigh, mons pubis) under ITN—tangential necrosectomy, covered with ASTSG (mesh ratio 1.5:1), dressed with a VAC-system for 5 days (Fig. 1c, d). Conservative treatment of the hands, genital and left thigh with PuF-Ag dressings. Custom-made burn pressure garments	2	18	3	The skin grafts were completely healed in place. All other burn wounds completely healed using conservative treatment. Hypertrophic scars, discoloration, and dysesthesia remained
3	54	Male	4.5	4	No	Upper thigh (left, IIb°), abdomen (IIa–IIb°), hand (left, IIa°)	Unknown	At the time of the explosion, the E‑cigarette was in the left front pants pocket and started to burn suddenly for no apparent reason	Arterial hypertension, arteriosclerosis	Initial treatment in an external trauma surgery clinic and transfer to our emergency room. The patient was admitted to the normal bed station. Close re-evaluations and dressing changes (PuF-Ag) were performed. Patient declined the recommended operation and continued outpatient treatment	0	7	0	Delayed wound healing along the abdominal wounds was present for a few months. Finally, all burn wounds completely healed using conservative treatment. Discoloration, visible scar formation (abdominal), and hypesthesia remained
4	40	Male	3.5	3	No	Upper thigh (left, I–IIa°), hands (both, I–IIa°)	Public	Explosion of the E‑cigarette, which the patient had been carrying in the left front pocket of the pants	None	Conservative outpatient treatment (dressing with fatty gauze, thereafter with PuF-Ag)	0	0	–	Complete healing
5	16	Male	4.5	3	Yes	Upper thigh (left, IIa–III° > right, I–IIa°), lower thigh (left, I–IIa°)	Unknown	The Li-ion battery of the E‑cigarette (carried in the left pants pocket) exploded without any warning	None	First presentation and inpatient admission to our clinic occurred on day 3 after accident. The patient underwent observation for five days and re-evaluation. The wounds evinced satisfactory healing tendencies. Surgery was not needed. Conservative treatment with PuF-Ag dressings was administered on an outpatient basis	0	5	0	Delayed wound healing along a small (1.5 × 2 cm) full-thickness damaged area on the left upper thigh. Healed under conservative therapy
6	59	Male	3	4	No	Face (I–IIb°), hand (left, IIa°)	At home	The E‑cigarette exploded during operation	COPD IV, N. bronchi	The patient refused the recommended inpatient treatment. Conservative outpatient treatment with PuF-Ag dressings and wound ointment was provided	0	0	–	Complete healing. Discoloration remained
7	54	Female	2	5	No	Upper thighs (both, I–IIa°), hands (right I–IIb° > left I–IIa°)	Public, Outdoor	The battery of the E‑cigarette exploded while being carried in the right jacket pocket	Diabetes mellitus type II	Conservative outpatient treatment with PuF-Ag dressings	0	0	–	Complete healing

Burn depth: I° superficial; IIa° superficial partial thickness; IIb° deep partial thickness; III° full thickness

*SA* Sedoanalgesia; *ICU* Intensive care unit; *PuF-Ag* Silver salt-containing polyurethane foam; *ASTSG* Autologous split-thickness skin grafts; *VAC* Vacuum-assisted closure; *ITN* Intubation narcosis

Of the patients two were female and five male, which corresponds to a female to male ratio of 2:5. The median patient age was 43 years, ranging from 16 to 59 years (range: 43). Due to the countable number of patients, non-parametric data were used. Patients suffered superficial to full thickness burn injuries with a median TBSA of 4.5% (range: 5.5) ranging from 2–9%. Of the patients three exhibited full thickness burn injuries but none of the patients suffered an inhalation injury. The median Abbreviated Burn Severity Index (ABSI) according to Tobiasen [22] yielded a value of 4 (range: 2), ranging from 3 to 5.

Of the patients 6 exhibited burn injuries along their upper thighs (6/7) and hands (6/7); additionally, 2 subjects suffered burns of the abdomen (2/7), 1 patient had partial thickness burns along the penis and scrotum (1/7) and 1 patient showed partial thickness burns of the face (1/7, case #6). None of the patients evinced concomitant injuries.

**Fig. 1:**
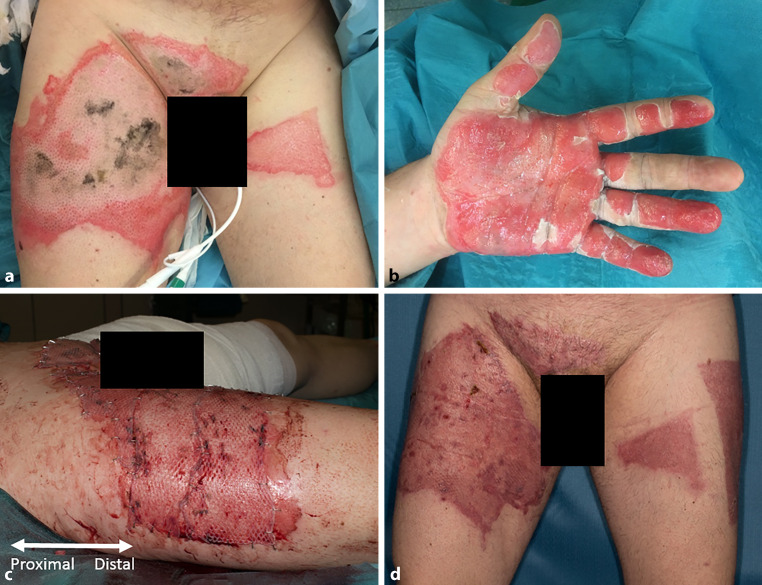
Case 1 on admission: upper thigh bilateral and mons pubis (**a**), left hand volar (**b**). Intraoperative: upper thigh right and mons pubis after necrosectomy and split skin transplantation (**c**); 13 days after surgery: upper thigh bilateral a mons pubis healed (**d**)

Only in one case (case #6) did the E‑cigarette explode during operation (1/7). In the other studied cases (6/7), the devices, i.e., the E‑cigarettes (3/6) or the Li-ion rechargeable batteries (3/6), were not in use and the devices were carried and stored in the victims’ pants (5/6) and jacket (1/6) pockets during the explosion. At least half (≥ 4/7) of these E‑cigarette-associated explosions occurred in public places. In 2 patient files, we could not find any information regarding the accidents, of the patients 4 had to be admitted (4/7), and 2 (Fig. 1) required intensive medical care extending longer than 24 h (2/7). These two patients required at least one surgical procedure because of the wide full thickness burns. The two subjects who were in need of surgery (2/7) underwent 3.5 (range: 3) operations (debridement 2/2, tangential necrosectomy and autologous split-thickness skin grafting 2/2) medially. The wounds of the other five patients (5/7) could only be treated conservatively with mainly polyurethane foam wound dressings containing silver salt and showed satisfactory results. In this study, no patient sustained major complications during the operation or in the postoperative course. The median length of inpatient (4/7) stay was 12.5 days (range: 26 days) in total, and the median length of ICU stay was 9 (range: 12) days for subjects who required intensive care (2/4). No patient died during the investigation period (Table 2).

**Table 2:** Results: Shows the evaluated characteristics

Characteristics		
		All patients (*n* = 7)
Sex distribution (female:male)		2:5
Age (years)		43 (r: 43)
Accident site (number)	Public	4 (57%)
	Unknown	2 (29%)
Device (number)	Not in use	6 (86%)
	Carried in pocket	6 (86%)
Full-thickness burn (number)		3 (43%)
Inhalation trauma (number)		0 (0%)
TBSA (%)		4.5 (r: 7)
ABSI (score)		4 (r: 2)
Affected body region (number)	Head and face	1 (14%)
	Thorax	0 (0%)
	Abdomen	2 (29%)
	Upper arm and forearm	0 (0%)
	Hand	6 (86%)
	Thigh	6 (86%)
	Lower leg and food	1 (14%)
	Back and buttocks	1 (14%)
	Genitals	1 (14%)
Need of operative treatment (number)		2 (50%)
Number of operations (*N* = 2) (number)		3.5 (r: 3)
Mortality (number)		0 (0%)
Hospitalization (number)		4 (57%)
Intensive care (number)		2 (29%)
Length of inpatient stay (days)	Total (*n* = 4)	12.5 (r:26)
	Length of ICU stay (*n* = 2)	9 (r:12)

Results in ratio, median (range), number (%)

*TBSA *Total body surface area, *ABSI* Abbreviated Burn Severity Index, *ICU* Intensive care unit

## Discussion

This study demonstrates very similar accident courses (devices were not in use) and similar affected and injured body sites (upper thigh, hand) in the majority of the patients (six of seven) treated by our department. Burns to the genitals and buttocks have also been observed; however, injuries to the dominant hand and genitals represent the greatest burden for patients. Notably, at least half of the accidents took place in public (for two patients, the course of the accident could not be determined retrospectively), and in six of the seven cases, the devices were carried and stored in the patient’s pants (*n* = 5) and jacket (*n* = 1) pockets during the explosion. The explosions occurred spontaneously and without prior notice in all cases, and in six cases, the victims’ pants ignited. The results of the present study are intended to underline the unpredictability of such dangerous E‑cigarette-related accidents and the sometimes severe injuries that can result from such explosions. The depth of burn injuries associated with E‑cigarette explosions presents a serious concern, especially due to the pattern of injuries observed. In our findings, E‑cigarette burns caused full thickness burns in anatomically unfavorable locations, which may complicate treatment and recovery. Notably, three out of seven patients suffered full thickness burns, particularly on the upper thigh. This could be attributed to the common habit of carrying E‑cigarette devices in pockets near the thigh region, which exposes this area to intense thermal injury when the device malfunctions. These burns can lead to severe scarring, functional impairment, and a higher risk of infection due to the depth and extent of tissue loss. In other body regions, such as the hands, genitals and face, burns were mostly superficial to partial thickness; however, these areas are also highly sensitive and functional, so even partial thickness burns can still lead to discomfort, potential complications and cosmetic concerns. This distribution highlights the need for targeted protective measures and further investigation into device safety to minimize such injuries, especially in these vulnerable areas.

The E‑cigarettes and Li-ion batteries can, in essence, overheat, catch fire or explode, causing an open fire in any situation, whether in transport, storage, charging, or operation [9, 13]. Seitz et al. and Serror et al. reported in their studies published in 2018 that the majority (65–80%) of E‑cigarette explosions occurred while the devices were being transported or stored in the victim’s pockets [11, 20]. These facts are also described in a review by the U.S. Fire Administration for the years 2009–2019 [3]. The burn injuries in our investigation were caused in most cases (86%, 6 of 7) by exploding E‑cigarettes or by the Li-ion batteries, which in each case were being worn and stored in the victims’ clothing. This supports the conjecture that it is primarily carrying or keeping these devices or their rechargeable batteries on one’s person that constitutes a nonnegligible potential risk for the occurrence of dangerous incidents. An assessment by the US Consumer Product Safety Commission (CPSC) for the years 2015–2017 further underlines this observation; it showed that the majority of patients after thermal incidents with E‑cigarettes mainly evince burns on the thighs and hands [1]. Specifically, transporting E‑cigarettes and rechargeable batteries in their own carrying cases or boxes can be recommended. This would first prevent the incidental formation of short circuits through contact with loose metal objects, such as key rings or coins in pockets, as well as prevent the accidental activation of the devices [9, 14, 23]. Second, should thermal runaway nevertheless occur, carrying the device in a case outside the clothing would inhibit direct contact with the body surface and could contribute to reducing the extent of any burns [9].

In accordance with the extent of burn injuries described in the literature, which is on average 2.5–5% TBSA, this study reported a comparable TBSA with a median of 4.5% [11, 18–20, 22].

Along with thermal and chemical injuries, the pressure wave caused by the explosion of an E‑cigarette can also lead to injuries that can be life-threatening [24, 25]. This pressure wave can instantly turn shattered pieces of the device into projectiles and severely injure teeth, facial bones, blood vessels, eyes, the brain or other organs [26–31]. In this context, two deaths caused by exploding E‑cigarettes have already been reported in the media [5, 32]; however, trauma resulting in death due to E‑cigarette explosions are exceptions. In our analysis, no patient suffered any concomitant trauma requiring treatment and no deaths were observed.

If such an incident were to occur in a crowd or on public transportation, for example, in an aircraft, the consequences for the consumer as well as surrounding persons could be fatal [33–35]. In 2022, E‑cigarettes were the main reason for battery explosions in airplanes [33]. Currently, the use and charging of E‑cigarettes on airplanes as well as the transportation of devices and their batteries in checked luggage are banned by the U.S. Federal Aviation Administration [36].

Recommendations to sell, buy and use only tested E‑cigarettes, rechargeable Li-ion batteries and components with national conformity, e.g., CE marking of European Conformity or UL Certification Mark in the USA, might prevent the circulation and use of deficient products or their modification with incompatible components and thus might also reduce the incidence of thermal injuries associated with E‑cigarettes [3, 4, 11, 23]. In view of the risk of explosion due to inadequate components of the devices, disposable vape pens could be considered a safer option as users might be less motivated to swap out or modify the components.

In addition, the establishment of a national, European or international register, e.g., the U.S. National Electronic Injury Surveillance System (NEISS) register [1], would be indicated for recording and monitoring incidents related to E‑cigarettes or Li-ion batteries. These data could then be used as an objective basis for future decisions to reduce such accidents.

## Conclusion

Explosions of E‑cigarettes often happen in public while the devices or rechargeable lithium-ion batteries are being carried in pants pockets, as shown in our study. This often ignites the victim’s clothing. Typical for these accidents are severe burns on the upper thighs and at least the dominant hands of the patients but burns on the genitals and buttocks have also been observed; however, injuries to the dominant hand and genitals represent the greatest burden for patients. In a crowd or on public transport, this fire could spread quickly and endanger surrounding persons. Due to the indisputable hazard to health and life caused by the fire or explosion of E‑cigarettes, it would be in the public interest to inform and raise awareness of these potential dangers among the general public and especially consumers.

To our knowledge, compared to the current literature, this work has the longest observation period and most published cases in the German-speaking regions (Austria, Germany, Switzerland), paired with a very detailed investigation of the accident courses and locations as well as the resulting burn patterns; however, injuries to the dominant hand and genitals represent the greatest burden for patients.

## Abbreviations

ABSI: Abbreviated Burn Severity Index
ASTSG: Autologous split-thickness skin grafts
E‑cigarette: Electronic cigarette
ICU: Intensive care unit
ITN: Intubation narcosis
PuF-Ag: Silver salt-containing polyurethane foams
SA: Sedoanalgesia
TBSA: Total body surface area
VAC: Vacuum-assisted closure
